# A rat model of complete atrioventricular block recapitulates clinical indices of bradycardia and provides a platform to test disease-modifying therapies

**DOI:** 10.1038/s41598-019-43300-9

**Published:** 2019-05-06

**Authors:** Nam Kyun Kim, David Wolfson, Natasha Fernandez, Minji Shin, Hee Cheol Cho

**Affiliations:** 1 Department of Pediatrics, Emory University, Atlanta, GA USA; 2 Department of Biomedical Engineering, Georgia Institute of Technology and Emory University, Atlanta, GA USA; 3 Department of Pediatrics, Yonsei University College of Medicine, Seoul, South Korea

**Keywords:** Arrhythmias, Arrhythmias

## Abstract

Complete atrioventricular block (CAVB) is a life-threatening arrhythmia. A small animal model of chronic CAVB that properly reflects clinical indices of bradycardia would accelerate the understanding of disease progression and pathophysiology, and the development of therapeutic strategies. We sought to develop a surgical model of CAVB in adult rats, which could recapitulate structural remodeling and arrhythmogenicity expected in chronic CAVB. Upon right thoracotomy, we delivered electrosurgical energy subepicardially via a thin needle into the atrioventricular node (AVN) region of adult rats to create complete AV block. The chronic CAVB animals developed dilated and hypertrophied ventricles with preserved systolic functions due to compensatory hemodynamic remodeling. Ventricular tachyarrhythmias, which are difficult to induce in the healthy rodent heart, could be induced upon programmed electrical stimulation in chronic CAVB rats and worsened when combined with β-adrenergic stimulation. Focal somatic gene transfer of *TBX18* to the left ventricular apex in the CAVB rats resulted in ectopic ventricular beats within days, achieving a de novo ventricular rate faster than the slow atrioventricular (AV) junctional escape rhythm observed in control CAVB animals. The model offers new opportunities to test therapeutic approaches to treat chronic and severe CAVB which have previously only been testable in large animal models.

## Introduction

Complete atrioventricular block (CAVB) is a major cardiac conduction disorder, in which the transmission of an electrical impulse from the atria to the ventricles is completely inhibited^1–3^. The subsequent slow AV junctional escape rhythm is inadequate to support CAVB patients’ hemodynamic needs, requiring the implantation of an electronic pacemaker device^4,5^. If left untreated, the ventricles of CAVB patients are susceptible to spontaneous and drug-induced QT prolongation^6,7^, which often precipitates to life-threatening arrhythmias such as torsades de pointes^8–10^. An animal model of CAVB would be ideal for studies aimed at understanding mechanisms of catastrophic ventricular tachyarrhythmias^11^, as well as developing therapies to mitigate arrhythmogenic events in CAVB^12,13^. We and others have developed large animal models of CAVB^12,14–17^, but those models are resource-exhausting and not widely available for standard benchtop research. Transgenic mouse models can exhibit spontaneous AV block^18,19^, but their degree and persistence of AV block are unpredictable, as CAVB is an adverse side effect of the transgene modification rather than a direct effect. Surgical models of CAVB have been documented in rodents^20–23^. However, we found these methods difficult to reproduce and adopt independently. One approach of injecting 70% ethanol into the AVN^20,24^, is hindered by a limited number of ablation attempts, as repeated doses of alcohol lead to global heart dysfunction and systemic complications, thus reducing survival. Other approaches require the use of custom-engineered, rodent-sized radiofrequency (RF) ablation catheters^22,23^, impeding widespread implementation in conventional laboratories.

We sought to develop a rodent model of CAVB that requires only a routine set of surgical instrumentation, yields a high success rate in 6-month old female rats, persists long-term (4 weeks), and recapitulates ventricular arrhythmias that are often manifested in CAVB patients^25^. Here, we present a long-term survival CAVB model in rats achieved via subepicardial delivery of electrosurgical energy into, and the subsequent fibrosis of, the AVN region. CAVB was achieved immediately upon ablation of the AVN and was stable for >4 weeks. The CAVB animals exhibited electrical abnormalities, including spontaneous and inducible ventricular tachyarrhythmias, and progressive ventricular remodeling, evidenced by increased stroke volume and ventricular hypertrophy with preserved ejection fraction at week 4. Notably, our chronic CAVB model revealed striking age and sex differences in survival rates, in which all males and 3-month old females died within the first 72 hours of creating CAVB, suggesting that the slow junctional escape rhythm is insufficient for supporting male and 3-month old female rats.

We also sought to reverse the severe CAVB-induced ventricular bradycardia by focal somatic gene transfer of *TBX18*, a concept we have previously demonstrated for converting chamber cardiomyocytes to induced pacemaker cells^12,26,27^. Direct intramyocardial delivery of *TBX18* into the left ventricular apex of chronic CAVB rats revealed emergence of ectopic ventricular beats at a rate higher than the slow AV junctional rhythm of the control animals. The *TBX18*-induced ventricular pacing is the first demonstration of the technique’s feasibility and disease-modifying potential, when the biologic is delivered after chronic CAVB had been established.

## Results

### Design of a custom, epicardial electrical ablation system

**Figure 1:**
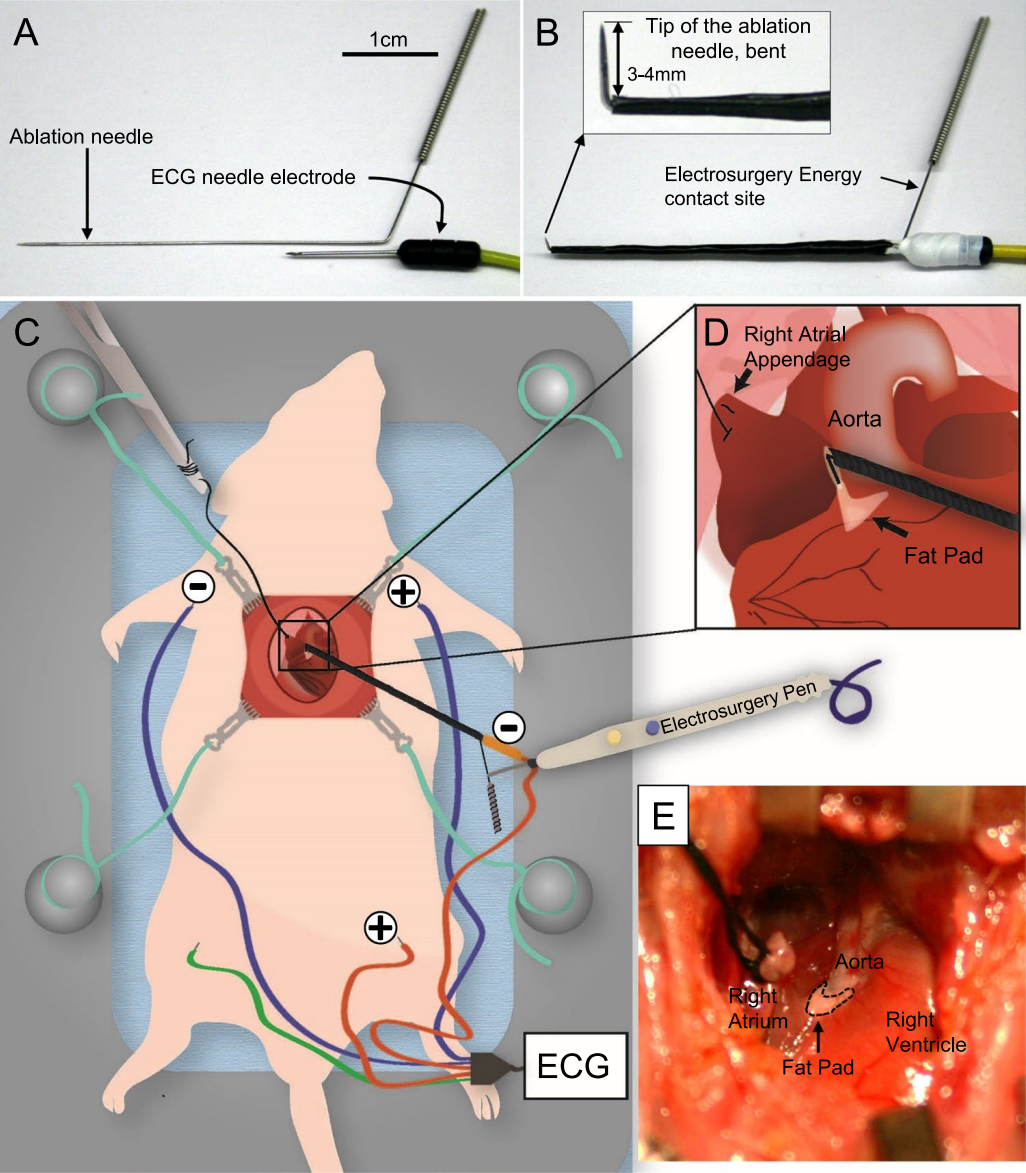
Design of electrosurgical needle ablation of the AV node. (**A**) An acupuncture needle and an ECG needle electrode, placed together to form a single unit to deliver electrosurgical energy and to record electrogram at the needle entry site. (**B**) The ablation needle and an ECG needle was assembled together with a polytetrafluoroethylene tape which also provides electrical insulation. The sharp end of the needle was bent at 3–4 mm from the end to limit the needle entry. (**C**) Illustration of the ablation needle, electrosurgical pen contact site, and the ECG electrodes during CAVB procedure. The intracardiac electrogram is recorded from the needle entry site (orange colored negative electrode) to left leg (orange colored positive electrode). (**D**) Needle entry is made through the anatomical landmark, fat pad, pointing toward the LV apex. (**E**) A photograph of the fat pad near the aortic root upon retracting the right atrial appendage.

We attempted to disrupt AVN conduction by focal ablation with electrical energy. We elected to use an electrosurgical unit over electrocautery, because electrosurgery delivers high-frequency electrical energy, traveling beyond the tissue-electrode contact site. On the other hand, electrocautery, which delivers heat only, is limited to burning tissues at the contact site. Initial trials of electrical energy delivery by direct contact of an electrosurgical pen on the AVN region caused an epicardial scar that remained largely superficial. This failed to generate reproducible CAVB. In order to deliver electrical energy subepicardially, we employed a thin needle of a 0.3 mm diameter (Fig. 1A). This needle was advanced through the epicardium into the AVN region, and was used as a conduit to deliver electrical energy to the AVN tissue. We also hypothesized that epicardial electrograms from the needle tip would show a sharp intracardiac potential wave which would be manifested near the P-wave if the needle entry was in proximity to the AVN. To test this, the ablation needle was coupled to a surface ECG electrode by wrapping them together with a polytetrafluoroethylene tape (Fig. 1B). This design enabled real-time monitoring of epicardial electrograms as the distal end of the ablation needle makes an entry into the AVN region (Fig. 1C,D). The distal end of the ablation needle was bent at 3 mm from the sharp end to control the depth of the needle entry (Fig. 1B), thereby preventing perforation to the surrounding myocardium and aorta. We determined the entry site of the ablation needle to the AVN area by the characteristic fat pad landmark consistently located between the aortic root and the medial wall of the right atrium (Fig. 1D,E, Supplemental video 1).

### Electrosurgical AVN ablation leads to stable CAVB in 6-month old female rats

**Figure 2:**
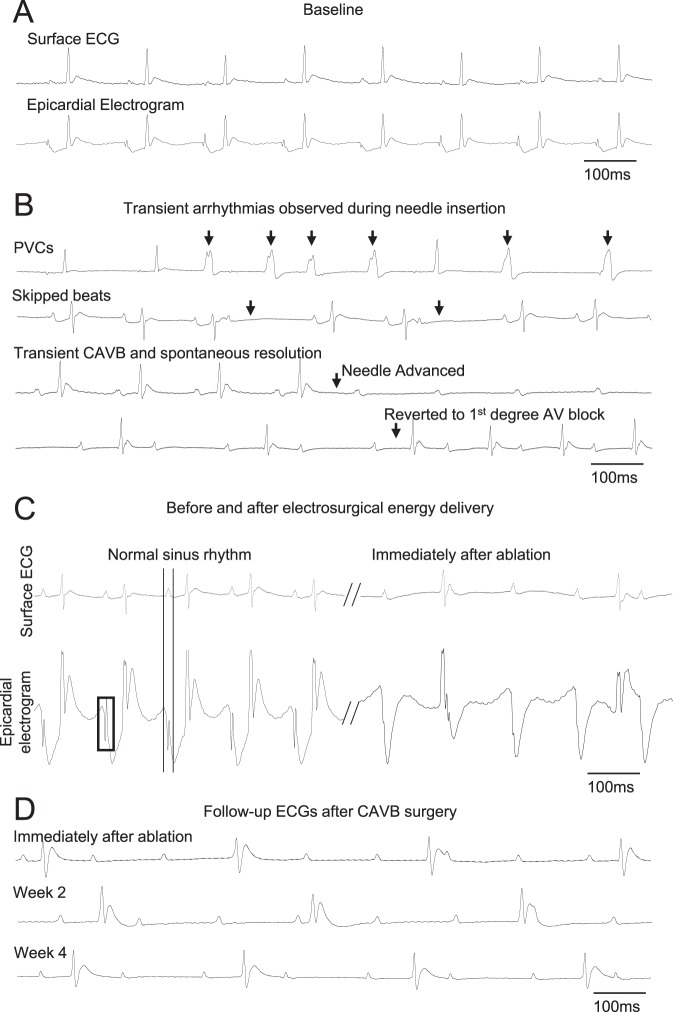
Surface and epicardial electrocardiograms before and after CAVB creation. (**A**) An example of surface ECG and simultaneous recording of epicardial electrogram under normal sinus rhythm. (**B**) Typical transient ventricular arrhythmias that could be observed during needle entry into the AVN region. (**C**) Upon the needle entry into the proper AVN region, sharp depolarizations starting within the P-wave from the epicardial electrode can be observed from the subepicardial electrogram. Upon successful CAVB, the His-like potentials are no longer observed. (**D**) Long-term follow-up of the CAVB animals demonstrate stable and complete heart block for at least 4 weeks.

**Figure 3:**
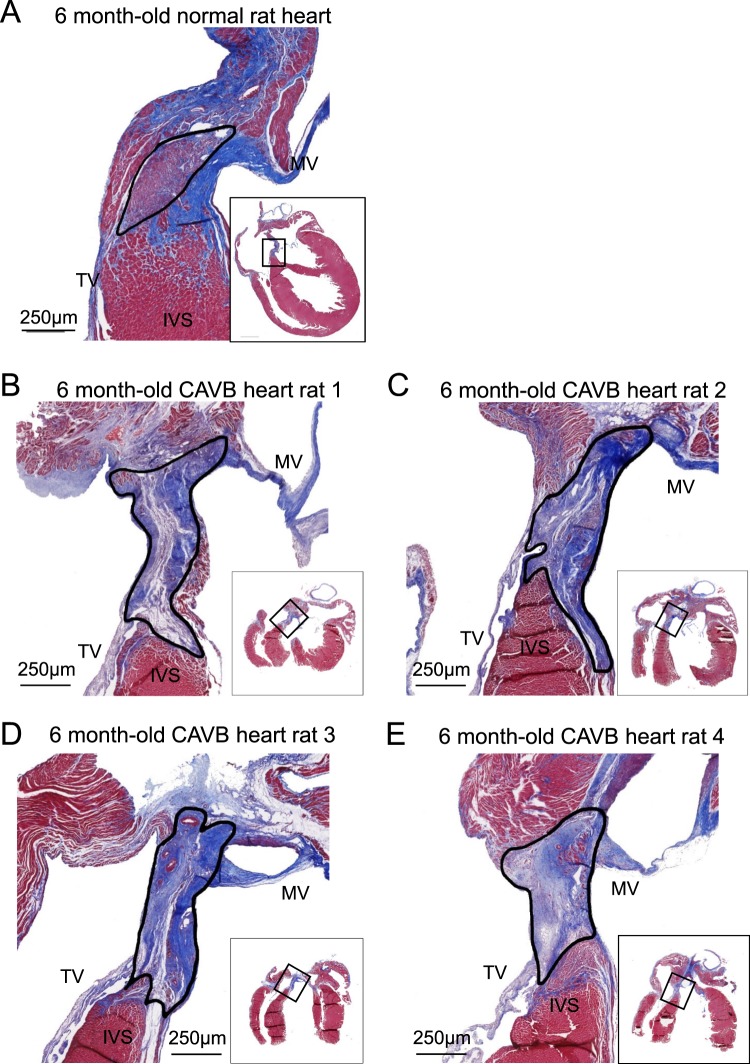
Extensive fibrosis in the AV nodal region 4 weeks after CAVB creation in 6-month old female rats. Masson’s Trichrome staining of the AVN area showing an increased presence of fibrotic tissue, (**B** to **E**, solid line) following CAVB surgery in four 6-months old female rats. The ablation site reveals extensive fibrosis compare with the normal rat heart (**A**, solid line) at 4-weeks post-CAVB creation procedure. The rats were 6-month old at the time of ablation and exhibited stable CAVB for 4-weeks post-surgery. (**A**) Histologic finding of healthy 6-months old female rat’s AV nodal area. (**B**–**E**) Representative histology 4-weeks post-ablation from 4 separate rats. The mean area of fibrotic region was 0.98 ± 0.15 mm^2^. MV, mitral valve; TV, tricuspid valve; IVS, interventricular septum.

**Figure 4:**
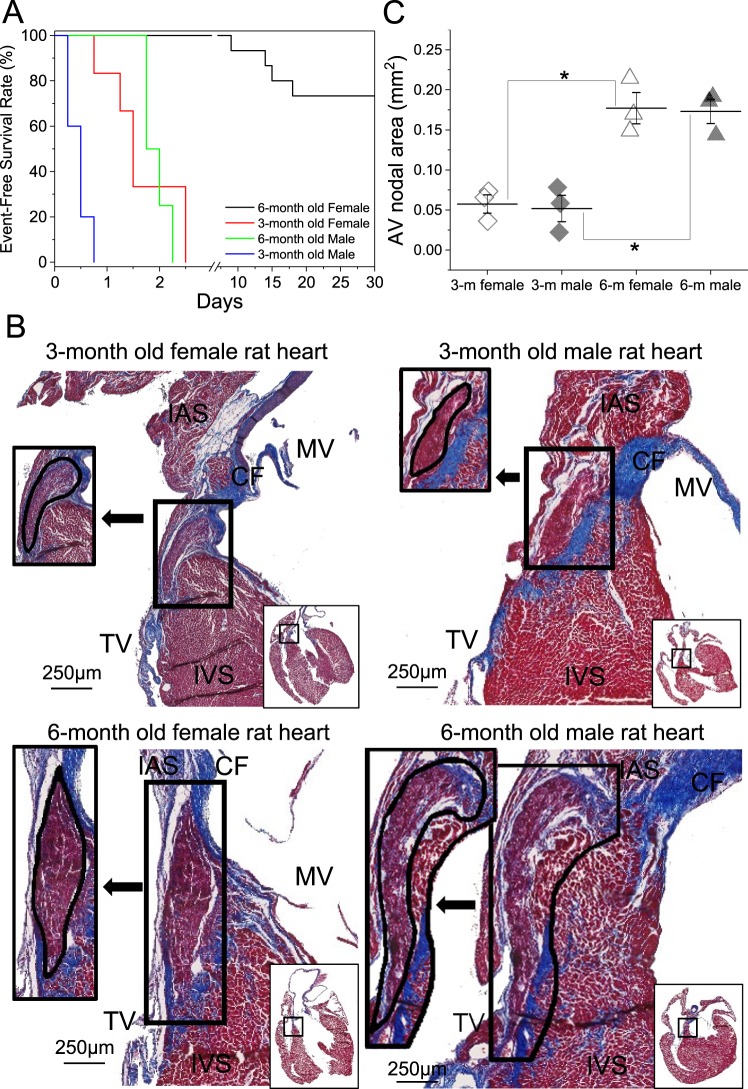
The 3-month old rats’ poor survival upon CAVB correlates with their relatively smaller compact AV nodal region. (**A**) Event-free survival curve of the CAVB rats illustrates the poor survival rates of the 3-month old rats and 6-month old male rats. (**B**) Representative images from the normal AVN region (solid line) of male and female rats at 3-month or 6-month of age. Compact nodal region adjacent to the central fibrous body is demarcated with a black line in an inset on the left of the arrow. (**C**) Mean area of the AV nodal region was similar between males and females within the same age group, but was significantly larger in 6-month old animals compared to 3-month old rats. CF, central fibrous body; IAS, interatrial septum; IVS, interventricular septum; MV, mitral valve; TV, tricuspid valve.

The following steps were taken prior to delivering electrosurgical energy: The bent distal tip of the ablation needle was advanced into the epicardial surface, posterior to the fat pad and posterolateral of the aorta, and pointing toward the left ventricular apex. This maneuver often elicited skipped ventricular beats, transient AV dissociations indicative of mechanical disruption of AVN or high His bundle conduction, and/or premature ventricular complexes (PVCs) (Fig. 2A,B and Table 1). Parallel to the surface ECGs, real-time epicardial electrograms at the needle insertion site revealed sharp depolarizations starting within the P-waves of the surface ECG (Fig. 2C). Electrosurgical energy was delivered upon satisfying these three criteria: (i) anatomical identification of the AVN fat pad, (ii) transient ventricular arrhythmias elicited by advancing and maneuvering the ablation needle, and (iii) sharp deflections on epicardial electrograms that coincided with P waves on the surface ECG (Supplemental video 2). An ablation power of 2 Watts was delivered for at most 60 seconds (Table 2). If stable CAVB was not observed after initial ablation, the power was increased to 3 Watts and ablation repeated for less than 10 seconds. After the second energy delivery, if persistent CAVB was still not observed, the needle was carefully retracted, repositioned to a new site matching the three criteria above, and the electrosurgical energy delivery was repeated. Successful CAVB induction was evidenced by complete dissociation of the QRS complexes from the P waves on bipolar limb leads (Fig. 2D). The thoracotomy was repaired when the CAVB was stable for 10–15 minutes. Histological examination of the AV junctional region 4 weeks after CAVB shows extensive fibrosis that left little viable myocardium in the AV junctional region (Fig. 3B–E, representative histology sections from four CAVB animals). Upon ablation of the AVN, the heart rate of adult female rats dropped immediately from a normal sinus rhythm of 351 ± 40 bpm to a junctional escape rhythm of 113 ± 37 bpm (n = 21). Fifteen out of the 21 (73%) 6-month old female rats were viable for ≥4 weeks with a stable CAVB (Fig. 4A). Three animals suffered sudden death soon after the survival surgery and one recovered from CAVB to 1° AV block. The mean junctional escape rhythm was stable throughout the 4 week follow-up at 122 ± 19 to 114 ± 22 bpm at week 1 to week 4 (Table 3).

**Table 1:** Spontaneous arrhythmias observed during entry of the ablation needle into the AV nodal subepicardial region.

Type of ventricular arrhythmias	Number of animals presenting the arrhythmias with some animals presenting more than one type of arrhythmias
Premature ventricular complexes (PVCs)	16/30
Skipped beats	11/30
Spontaneous AV dissociations	5/30

**Table 2:** Procedural Outcomes.

Parameters	
Procedure time (min)	85 ± 30
Recovery time (min)	23 ± 9
Ablative energy delivered (W)	2–3
Number of ablation attempts	3 ± 2
Estimated blood loss (mL)	0.9 ± 0.6

**Table 3:** Heart rate change during procedure and follow-up period.

	Atrial rate (bpm ± SD)	Ventricular rate (bpm ± SD)	Sample size (n)
Baseline heart rate	352 ± 40	352 ± 40	24
Immediately after CAVB	337 ± 38	102 ± 38	
Male rats	317 ± 31	83 ± 24	
3-month old	333 ± 28	65 ± 9	5
6-month old	291 ± 15	106 ± 15	4
Female rats	349 ± 38	113 ± 37	
3-month old	384 ± 24	84 ± 7	4
6-month old	360 ± 34	123 ± 39	11
1 week after CAVB	357 ± 47	122 ± 19 (females only)	11
4 weeks after CAVB	326 ± 39	114 ± 22 (females only)	8

### Male rats and 3-month old female rats are not viable after CAVB

Age-matched, 6-month old male rats were not viable after CAVB, all expiring within 3 days after the surgery (n = 4 rats). Immediately upon CAVB, the ventricular rate of the 6-month old male rats was 106 ± 15 bpm, statistically similar to that of the 6-month old female rats at 123 ± 39 bpm (*p* = 0.49). However, the male rats weighed significantly more at 504 ± 15 g compared to the age-matched female rats weighing 372 ± 63 g (*p* < 0.05, Table 4). We performed CAVB on 3-month old male rats (n = 5 rats) size-matched to the 6-month old female rats, but all five 3-month old male rats expired as well within a day of creating CAVB. The ventricular escape rate upon CAVB induction was significantly slower in the 3-month old male rats at 65 ± 9 bpm than the 6-month old males (106 ± 15 bpm, *p* < 0.05). We asked if the mortality in the males was strictly due to sex difference, and performed CAVB in 3-month old female rats weighing 271 ± 16 g. The 3 month old female rats also expired in 72 hours after CAVB induction (Fig. 4A), exhibiting a mean ventricular rate of 84 ± 7 bpm (n = 4 rats), that was statistically higher than the age-matched 3-month old male rats (65 ± 9 bpm, *p* < 0.05), but slower than the 6-month old female rats (123 ± 39 bpm, *p* < 0.05). Subcutaneous injection of isoproterenol during the post-op recovery period transiently increased the slow junctional rhythm of the 3-month old animals, but did not aid in the survival of those animals beyond three days post-op. To learn if the differences in the acute survival rates CAVB animals may be due to developmental stages rather than to sex difference *per se*, we examined the normal compact AVN areas near the central fibrous body of 6-month old female rats, 6-month old male rats, 3-month old female rats, and 3-month old male rats (n = 3 for each group). The compact AVN region near the central fibrous body was distinctively smaller in the 3-month old rats of both sexes compared to that of the 6-month old rats (Fig. 4B,C). In female rats, the mean area of the compact AV node was 0.057 ± 0.020 mm^2^ in 3-month old, compared to 0.173 ± 0.026 mm^2^ in 6-months old females (n = 3 rats, *p* < 0.05). Likewise in male rats, the mean area of the AVN was 0.052 ± 0.028 mm^2^ in 3-month old, compared to 0.177 ± 0.034 mm^2^ in 6-month old males (n = 3 rats, *p* < 0.05). No significant difference was found in AVN area between age-matched male and female rats (Fig. 4C). These anatomical differences between the 3-month old and 6-month old rats are in line with the gradual postnatal expansion of the AVN region in humans^28^.

**Table 4:** Baseline Characteristics of Each Group.

Parameters	Female rats, 3-month old (n = 4)	Female rats, 6-month old (n = 11)	Male rats, 3-month old (n = 5)	Male rats, 6-month old (n = 4)
Age (days)	95 ± 7	188 ± 9	93 ± 1	183 ± 8
Weight (g)	271 ± 16	372 ± 63	352 ± 24	504 ± 15

### Chronic CAVB leads to ventricular remodeling and increased arrhythmogenicity

**Figure 5:**
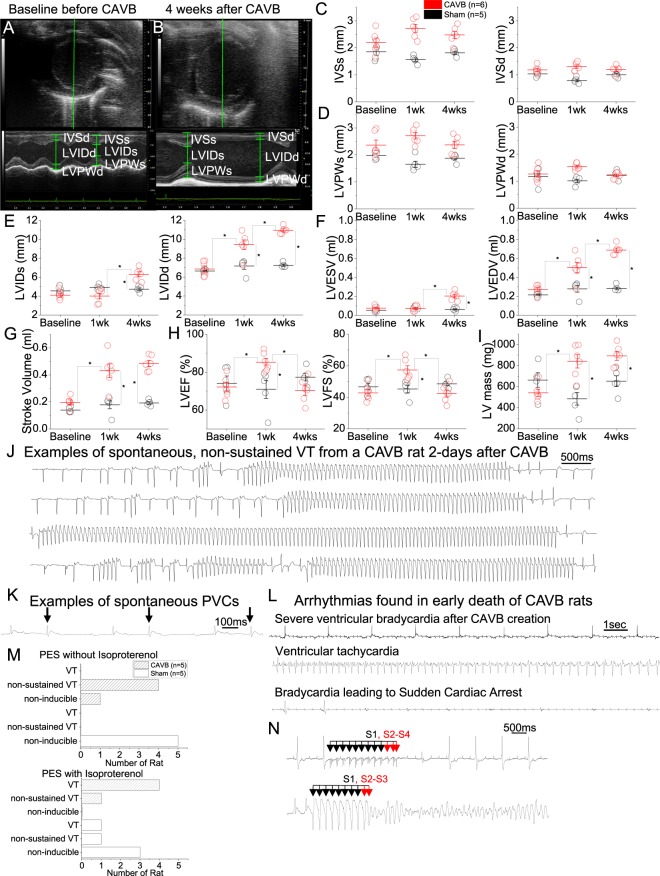
Echocardiographic findings before and after CAVB creation. (**A**) A parasternal short axis view (top) and M-mode (bottom) of normal sinus rhythm rat prior to CAVB surgery. (**B**) A parasternal short axis view (top) and M-mode (bottom) of the same rat at one month after CAVB surgery. At one month after CAVB, the left ventricle exhibited severe enlargement compared to the baseline. Mean data from 6 rats are analyzed to compare hemodynamic functions before and one month after CAVB: systolic and diastolic interventricular septum (**C**), LV posterior wall thickness (**D**), LV internal diameter (**E**), LV end systolic volume and end diastolic volume (**F**), LV stroke volume (**G**), LV ejection fraction and LV fractional shortening (**H**), LV mass (**I**). ECG telemetry revealed incidences of spontaneous, non-sustained ventricular tachycardia (**J**) as well as frequent PVCs (**K**) during first few days after CAVB surgery. (**L**) Representative ECG finding after CAVB creation in 3-month old rats. Following AVN ablation, severe ventricular bradycardia was observed (top), followed by non-sustained ventricular tachyarrhythmia (middle), and eventually sudden cardiac arrest (bottom). (**M**) Ventricular arrhythmia inducibility of complete AV block and sham-operated rats. In four out of five CAVB rats, programmed electrical stimulation (PES) induced non-sustained ventricular tachycardia (VT) which degenerated into sustained, polymorphic VT or ventricular fibrillation (VF) upon injection of isoproterenol. In one of the five CAVB rats, VT was induced only when PES was combined with isoproterenol injection. Ventricular arrhythmias were inducible in sham-operated animals only upon isoproterenol injection. One rat exhibited VT and another rat showed non-sustained VT upon PES with isoproterenol injection. Raw traces of PES-non-induced CAVB (top), and PES with isoproterenol-induced VF (bottom) are shown (**N**).

Unresolved, severe ventricular bradycardia is known to trigger compensatory ventricular remodeling and increased arrhythmogenicity^6,8,9,23^. We performed echocardiographic examinations over a 4-week follow-up period in CAVB animals and compared the data with sham-operated animals which underwent the same surgical procedures as the CAVB animals, including thoracotomy and advancement of the ablation needle but without delivering the ablative energy. Parasternal short axis view and M-mode echocardiograms at baseline just prior to CAVB induction (Fig. 5A) and four weeks after CAVB (Fig. 5B) in the same rat illustrates severely enlarged left ventricle at 4-week. As expected, the sham-operated animals (n = 5) showed no changes in interventricular septum (IVS) or left ventricular posterior wall (LVPW) dimensions at systole or diastole over the four-week follow-up period. Similarly, CAVB animals (n = 6) showed no significant changes from the baseline measurements to 1- and 4-week after CAVB in IVS or LVPW dimensions although the measurements showed higher trend compared to those of the sham-operated animals at 1- and 4-week time points (Fig. 5C,D). In contrast, left ventricular internal diameter (LVID) at systole increased at 4-week and diastolic LVID increased at 1-week and further more at 4-week in CAVB animals while those of the sham-operated rats remained the same (Fig. 5E). The increases in the LVID led to parallel increases in the end-diastolic and end-systolic left ventricular volumes (LVEDV and LVESV) over time in CAVB animals (Fig. 5F). Left ventricular stroke volume increased by >2-fold in CAVB animals at week 4 compared to baseline and to sham controls (*p* < 0.05, Fig. 5G). The LV ejection fraction and fractional shortening (LVEF and LVFS) increased transiently at 1 week after the surgery, but returned to the baseline levels by week 4 (Fig. 5H). The structural remodeling resulted in increased left ventricular mass by 299 ± 195 mg at week 1 and by 350 ± 94 mg at week 4 from the baseline LV mass of 542 ± 74 mg (Fig. 5I). All reported masses were approximated using echocardiography measurements. These results demonstrate that compensatory left ventricular remodeling ensues as the severe ventricular bradycardia transitions to a chronic condition in CAVB rats.

We examined for spontaneous ventricular arrhythmias by implanting an ECG telemetry device in three of the 6-month old female CAVB rats by 24/7 ambulatory telemetry. Spontaneous ventricular arrhythmias such as non-sustained ventricular tachyarrhythmias (NSVT) or PVCs (Fig. 5J,K) were observed in one of three CAVB rats two days after the AVN ablation. PVCs were more pronounced during the first two days after CAVB and resolved in 3–4 days. We examined arrhythmia inducibility with programmed electrical stimulation (PES) at week 4 after creating CAVB. PES elicited self-terminating, non-sustained VTs in 4 out of 5 CAVB animals. Upon administration of a β-adrenergic agonist, isoproterenol, PES-induced arrhythmias precipitated into sustained VT and/or ventricular fibrillation (VF) in the same four animals that initially exhibited non-sustained VT with PES only (Fig. 5M,N). The one animal that did not show VT upon PES alone degenerated to non-sustained VT when PES was performed upon i.p. injection of isoproterenol (5 mg/kg body weight). Sham-operated animals could not be induced into arrhythmia upon PES (Fig. 5M, upper panel). Upon injection of isoproterenol, PES led to non-sustained VT in 1/5 and sustained VT in another 1/5 sham-operated animal (Fig. 5M, lower panel). These data indicate that CAVB increases susceptibility to ventricular arrhythmias in rats that are resistant to arrhythmias under normal heart function^29,30^.

### The CAVB model uncovers emergence of a distinct ventricular pacing rate induced by direct myocardial gene transfer of TBX18 *in vivo*

**Figure 6:**
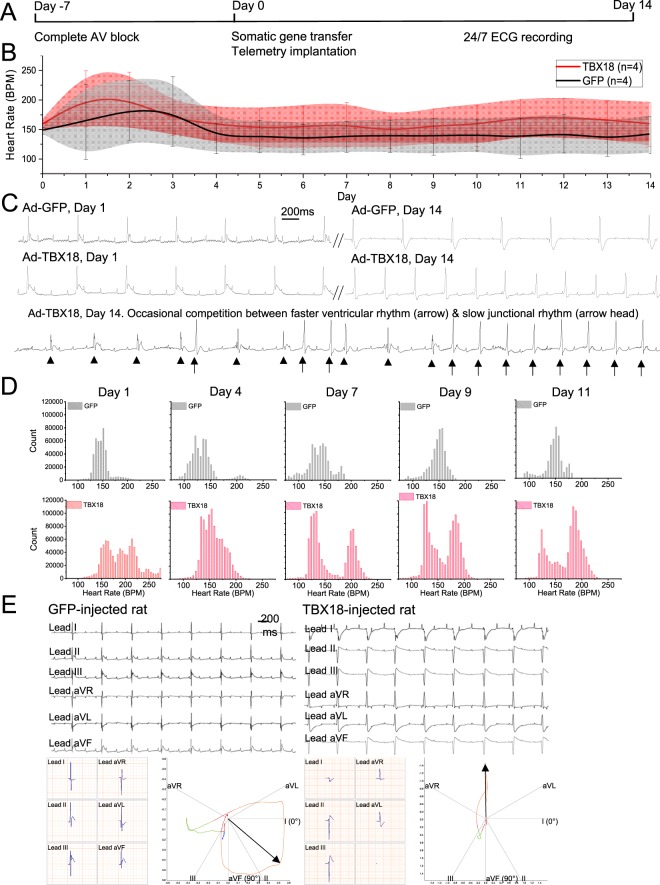
Focal *TBX18* gene transfer to the left ventricular apex of CAVB rats creates ventricular pacing that is faster than the slow junctional rate. (**A**) Timeline of the study indicating creation of CAVB and confirmation of chronic and stable CAVB for 7 days, second thoracotomy and focal injection of Adeno-*TBX18* at the left ventricular apex, and a 14-day recording of ECG telemetry. (**B**) Mean heart rates over 2 weeks of *TBX18*- or GFP-injected animals. Shaded areas indicate the standard deviation of the heart rate at each time point. (**C**) Surface ECGs from GFP- (top) or *TBX18*-injected rat (middle) obtained at the time of and 2 weeks after gene delivery. *TBX18*-injected animals often exhibited two competing ventricular rhythms, one presumably from the slow junctional rhythm (arrowhead) and the other due to *TBX18*-injection (arrows). (**D**) Heart rate histograms of *TBX18*-injected animals show that a second major peak emerges 7 days post gene delivery, which is faster than the slow junctional rhythm. (**E**) Cardiac axis mapping of GFP-injected (left) and *TBX18*-injected (right) rats at 7 days post-injection. The faster ventricular rhythm in *TBX18*-injected animals exhibits QRS axis change and wider QRS complexes, which indicate retrograde conduction and myocardial depolarization that propagated without the ventricular conduction system, respectively.

We have previously demonstrated that re-expression of an embryonic transcription factor, *TBX18*, converts chamber cardiomyocytes to induced-pacemaker cells^12,26,27^. Focal delivery of *TBX18* into the ventricular myocardium gave rise to ectopic ventricular beats in both small^27^ and large^12^ animal models, providing evidence for a transcription factor-based biological pacemaker. Although our porcine model of CAVB closely mimicked clinical CAVB^12,31^, our previous small animal model of ventricular bradycardia relied on transient AV dissociation resulting from non-specific, cholinergic suppression of AV conduction in anesthetized rodents^27,32^. Using the current rat model of chronic CAVB, we focally delivered an adenoviral vector expressing *TBX18* (Adeno-TBX18) into the left ventricular apex of 6-month old female rats upon confirming stable and complete AV block at 1 week after ablation surgery (Fig. 6A). When the heart rates from the ambulatory CAVB animals were averaged each day, the *TBX18*-injected animals exhibited a mean heart rate that trended higher than the ventricular rate of the GFP (Adeno-GFP) control animals (n = 4 for control and for TBX18-injected group, Fig. 6B) throughout the 2 week study period. The heart rate fluctuated widely within a given 24 hour analysis window, contributing to the large standard deviations in both groups (Fig. 6B, shaded areas). Closer examination of the telemetry data indicated that the control, Adeno-GFP injected CAVB animals’ ECGs were fairly uniform, with slow junctional rhythm pacing the ventricles. In contrast, *TBX18*-injected animals showed episodes of faster ventricular rhythm and PVCs that appeared to be competing with the slow junctional rhythm (Fig. 6C). To quantify the distribution of ventricular beating rates, we plotted beating rate histograms upon creating CAVB (Supplementary Fig. 1F), and then after delivering either GFP or *TBX18* (Fig. 6D). After CAVB, but before the second thoracotomy and gene transfer, the heart rate histogram showed a single-peak distribution throughout a 9-day follow-up (n = 3 rats, Supplementary Fig. 1F). A second thoracotomy with Adeno-GFP delivery did not alter the heart rate histogram with a single modal heart rate that started at 158 ± 31 bpm on day 1 after the gene delivery and stabilized at lower ventricular rates of 137 ± 26, 137 ± 31, and 135 ± 34 bpm on days 7, 9, and 11 respectively (n = 4 rats, Fig. 6D top). Interestingly on day 4 after Adeno-GFP delivery, the heart rate histogram contained a small bimodal distribution but is not present in later time points. In contrast, the heart rate histograms from *TBX18*-injected animals (TBX18) resolved two distinct peaks in the heart rates that lasted beyond 1 week post-delivery; one peak at a rate similar to the slow junctional rhythm of the control animals, and a second peak at a faster ventricular rate (n = 4 rats, Fig. 6D, bottom). We posit that the faster ventricular beats originated from the *TBX18*-injected site while the slow junctional rate originated from the His bundle. We recorded 6-lead surface ECGs under anesthesia to examine the cardiac axis of ventricular propagation. The slow junctional rhythm from Adeno-GFP injected animals corresponded to antegrade conduction on vector cardiogram analysis (Fig. 6E, left panels). In contrast, ventricular beats in *TBX18*-injected rats resulted in retrograde conduction, which is in line with *de novo* biological pacing at the left ventricular apex (Fig. 6E, right panels). Additionally, we observed wider QRS complexes in *TBX18*-injected animals compared to GFP control animals, in line with the expected slower ventricular propagation from apical pacing. Thus, our rat model of chronic CAVB enabled assessment of the ventricular pacing originating from *TBX18*-induced pacemakers in relation to the intrinsic AV junctional escape rhythm. To reduce invasiveness of two successive thoracotomies, we tested feasibility of replacing the second thoracotomy for gene delivery with echocardiography-guided, trans-thoracic injection of biologics. Echo-guided injection of Adeno-mCherry into the left ventricular myocardium of an anesthetized rat generated focal, intramyocardial mCherry expression, negating the need for thoracotomy (Supplementary Fig. 1A–E).

## Discussion

Previous approaches to creating CAVB in rodents relied on injection of cytotoxic chemicals^20,33^, use of custom-made radiofrequency ablation catheters^6,23^, or delivering heat by electrocautery to the AVN area^20,21^. The epicardial fat pad served as the anatomical landmark for the AVN region in all those models. However, because the location of the AVN can vary significantly from animal to animal^34,35^, this anatomical landmark alone resulted in limited success. We first attempted alcohol ablation of the AVN by direct, intramyocardial delivery of 70% ethanol to the AVN region^20^. This elicited mostly 1° or 2° AV blocks, or a 3° block that spontaneously reverted back to normal conduction within a few minutes. Repeat injections of 70% ethanol led to global damage to the myocardium, coagulation in the ventricular cavities, and eventual mortality. This method was abandoned.

Our technique delivers high frequency electrical energy via a sharp needle to ablate and necrotize the target tissue in a focal manner. We added simultaneous intracardiac recording function^33^ by attaching an ECG electrode to the proximal end of the ablation needle, which allowed real-time recording of subepicardial electrogram as the needle is advanced into the AVN region. This subepicardial electrogram (Fig. 2A, lower panel) revealed sharp peaks, which coincided with the P-waves during sinus rhythm but disappeared upon CAVB (Fig. 2C). Intracardiac recording of His bundle signals from a bipolar electrode would normally follow the P waves and precede the QRS waves^6^. Thus, the sharp potentials on the epicardial electrogram (Fig. 2C, lower panel) before ablation are indicative of electrical potential from AV nodal area.

It is notable that the 6-month old female rats could survive CAVB creation with a slow junctional rate at approximately 1/3 of their normal sinus rhythm. In a rabbit model of CAVB, the survival of the animal was highly dependent on the implantation of an electronic pacemaker, programmed to pace at >50% of the rabbit’s normal sinus rhythm^33^. In contrast to the hardy female rats, no 6-month old male rats survived the first 72 hours post-CAVB creation. We first hypothesized this difference in survival to be due to the male’s larger body weight and surface area. To address this, male rats were body weight-matched to the 6-month old female rats, and consequently, were three months younger than the females (93 ± 1 vs. 188 ± 9 days old, respectively). Surprisingly these younger, smaller male rats showed a significantly higher mortality rate compared to their older counterparts (0% survival in 24 hours), indicating an additional age-dependent effect on mortality. Further investigation showed a similarly increased mortality rate in 3-month female rats with CAVB (0% survival in 2.5 days). In both sexes, 3-month old rats were shown to die of ventricular bradycardia and sudden cardiac arrest (Fig. 5L), leading us to believe that CAVB rat mortality is highly dependent on the heart rate immediately following AV node ablation. In both sexes of 3-month old rats with CAVB, the ventricular rate was significantly slower (males 65 ± 9 bpm; females 84 ± 7 bpm), compared to their older 6-month old counterparts (males 106 ± 15 bpm; females 123 ± 39 bpm), suggesting that the ventricular conduction system may further develop as the rats age. Our histology data support this notion; the compact AVN area of 3-month old rats is much smaller than that of 6-month old rats (Fig. 4B,C). In line with our data, the human compact AVN develops extensions towards the high His bundle and undergoes widening of the transitional cell area in an age-dependent manner^28^. Younger rat (male and female) hearts containing a smaller AV nodal area, have significantly slower ventricular rates than their older counterparts following AV ablation. As shown in the ECG data (Fig. 5L), this severe bradycardia is lethal and leads to cardiac arrest. Whereas, 6-month old rats have more elongated AV nodal areas and higher ventricular rates following ablation, which help to prolong their initial survival. Following CAVB creation, the ventricular rate of 6-month old males was comparable to that of the 6-month old females, yet 2 days following ablation, the survival rate of male 6-month old CAVB drops significantly. Further investigation and future studies are needed to explain this phenomenon. We hypothesize it is due to the lower cardiac index of the larger, heavier male rats compared to females; the slow escape rhythm upon CAVB creation is inadequate to sustain the 6-month old male rats due to their larger body mass.

Our rat model of chronic CAVB faithfully recapitulated the anticipated disease course involving arrhythmogenicity and compensatory hemodynamic remodeling. During the first few days post-op, PVCs and/or skipped beats were frequently observed as well as non-sustained ventricular tachyarrhythmias which likely precipitated into lethal arrhythmias and contributed to the post-op mortality. Spontaneous ventricular tachyarrhythmias became less frequent over time and were rarely sighted one week post-surgery. However a degree of arrhythmogenicity remained after 4 weeks post-ablation, as evidenced by the non-sustained and sustained VT/VF induced with PES. The severe ventricular bradycardia in the CAVB rats led to an immediate increase in the stroke volume (Fig. 5G), likely to compensate for reduced cardiac output^1,36^. Echocardiograms revealed structural remodeling of the left ventricle over 4 weeks; end diastolic and systolic volumes progressively increased over 4 weeks, leading to >2-fold increase in the LV mass. Interestingly, fractional shortening and the ejection fraction increased during the first week post-surgery, but returned to near baseline levels at 4 weeks post-surgery. Noting that congestive heart failure develops in dogs with chronic CAVB^37^, it is plausible that our rat model of chronic CAVB may also develop congestive heart failure with preserved ejection fraction^38^. This warrants further investigation.

We and others have previously presented gene- and/or cell-based ventricular pacing as alternatives to electronic pacing devices for patients suffering from symptomatic ventricular bradycardia^12,27,39,40^. Motivation for hardware-free cardiac pacing goes beyond adult populations with device-related complications^41,42^. Current device-based cardiac pacing is incompatible for pediatric patients with congenital heart defects who are small yet growing, and the incidence of complications due to indwelling hardware are increasing^43–45^. Although we have demonstrated proof-of-principle for biological cardiac pacing, previous small animal models were limited to brief heart block caused by pharmacologic suppression of the cardiac conduction system. We took advantage of our rat model of chronic CAVB to test gene-based cardiac pacing by focal delivery of an adenoviral vector expressing *TBX18* to the left ventricular apex of the rat heart one week after creating the CAVB. Re-expression of *TBX18*, a transcription factor that is essential for proper embryonic development of the SAN^46,47^, induces automaticity in postnatal ventricular cardiomyocytes *in vitro* and ventricular pacing *in vivo*^12,27^. Analysis of their ambulatory ECG over a 2 week period revealed that a ventricular rate >200 bpm emerged by day 7 post-gene delivery in *TBX18*-injected animals, which was distinct from and faster than the slow junctional rhythm (Fig. 6C). To visualize this distinct beating pattern, beat-beat heart rate histograms revealed a bimodal distribution of heart rates in the *TBX18* animals, indicative of two competing pacing sites (Fig. 6D). These data indicate that *TBX18* creates a new ventricular pacing site which begins to compete with the native junctional rhythm in the same animal.

Taken together, the rat model of chronic CAVB presented here is conducive to gaining mechanistic insights into severe ventricular bradycardia and its subsequent degenerative processes. It also offers new opportunities for developing therapeutic modalities for mitigating the disease in a small animal model. Particularly, future studies on biological pacemakers can now quantitatively assess their robustness, longevity, autonomic regulation and arrhythmogenic risks in ambulatory rodents with chronic CAVB.

## Methods

All surgical procedures and animal care protocols were in adherence and approval of the IACUC of the Emory University School of Medicine.

### Surgical procedures

Sprague Dawley rats (Charles River Laboratories International, Inc., Wilmington, MA) were anesthetized in an induction chamber with 5% isoflurane in 100% oxygen at a constant flow rate of 2 L/min for 6 minutes. Endotracheal intubation was performed with a 14-gauge, 57 mm long intravenous catheter. The animals were ventilated with a respirator (model 683, Harvard Apparatus, South Natick, MA) at 85 respirations per minute with a 2.5 ml stroke volume. Anesthesia was maintained in 2% isoflurane mixed with 100% oxygen throughout the surgery. Sterile lubricating eye ointment (LubriFresh^TM^ P.M., MAJOR^®^ Pharmaceuticals, Livonia, MI) was applied following induction of anesthesia to prevent corneal drying. Monopolar electrosurgical current was delivered to the AVN region via an active electrode connected to a high frequency electrosurgical generator in a coagulation mode (Aaron 950, Bovie Medical, Clearwater, FL). A reusable dispersive grounding pad electrode (DRE Veterinary, Louisville, KY), serving as the return electrode, was placed on the back of the rat. The back of the rat was shaved to minimize electrical resistance between the animal and grounding pad. We found that a larger surface area grounding pad reduced the resistance and increased electrical current, thereby reducing the voltage necessary to achieve the same delivered power. Smaller ground pads or poor contact between the skin and the return electrode significantly reduced the effectiveness of AVN ablation and generated excessive heat at the skin-ground pad contact site, leading to severe skin burns. In order to ensure a sufficient skin-ground pad interface, we found that application of ultrasound electrode gel between the skin and ground pad significantly increased the effectiveness of electrosurgical AVN ablation. During surgery, body temperature was maintained at 37 °C with a water blanket (T/Pump Professional, Stryker, Kalamazoo, MI). Carprofen (5 mg/kg) and Buprenorphine SR (1 mg/kg) were given subcutaneously for analgesia. Atropine (0.05 mg/kg) was given intramuscularly to reduce pulmonary secretion. Upon ablation of the AVN, the thoracic cage was repaired with a 4-0 polyglycolic acid absorbable suture (SJ496G, McKESSON, Alpharetta, GA) and the lungs were fully inflated for two seconds to avoid pneumothorax immediately before closing the thoracic cavity. External thoracic muscles were repaired layer by layer with 4-0 absorbable suture. Skin was sutured with 4-0 monofilament polypropylene non-absorbable suture (P8683-SP, MYCO medical, Apex, NC). The isoflurane ventilation was reduced to zero after completion of the surgery. Once spontaneous breathing was evident, the endotracheal tube was removed. If excessive blood loss occurred (>2 mL), 2 mL of 0.9% sodium chloride solution was administered subcutaneously. Carprofen (5 mg/kg) was administered subcutaneously as a post-surgical analgesic at 24, 48, and 72 hours after surgery.

### Sham control group

Five Sprague Dawley rats underwent sham operation. Baseline echocardiogram was performed one day before sham operation. Sham operation was identical to CAVB surgery including right thoracotomy and advancing of the ablation needle into the AVN, but without delivering the electrosurgical energy. Echocardiogram and programmed electrical stimulation were performed at analogous time points as in CAVB rats.

### Real-time recording of surface electrocardiograms and epicardial electrograms during AVN ablation

Biopolar, limb-lead surface electrocardiograms (ECG) were recorded on anesthetized animals with a BioAmp (FE135) signal amplifier, a PowerLab 8/35 digitizer and analyzed with LabChart Pro (ADInstruments, Colorado Springs, CO). Baseline ECGs were acquired just after intubation under anesthesia with 2% isoflurane. A partial right thoracotomy was performed in the third intercostal space to expose the right atrium and aorta. After retracting the thymus and removing pericardium, the right atrial appendage was partially ligated and the right atrium laterally retracted so as to avoid excessive tissue damage or bleeding during ablation. This maneuver exposed the epicardial aspect of atrioventricular nodal area, landmarked by the characteristic fat pad consistently located between the aortic root and the medial wall of the right atrium. The negative electrode for lead II was replaced with the electrode connected to the ablation needle for epicardial electrogram. This was achieved by attaching the electrode of a surface ECG cable (MLA1203, ADInstrument) to the proximal end of a 0.3 mm diameter, 5 cm long ablation needle (DB106, DongBang Acupuncture, Korea, Fig. 1A) and insulating them together with polytetrafluoroethylene tape. 3 mm from the distal end (sharp end) were left exposed and bent at a 90 degree angle. Advancing the distal end of the ablation needle into the AVN region allowed real-time monitoring of subepicardial electrogram at the ablation site. The ablation needle, in contact with the ECG electrode, was painted with a liquid electrical tape (LTB-400, Gardner Bender, Menomonee Falls, WI) for electrical insulation, exposing only the sharp, distal 3–4 mm end of the needle (Fig. 1B,C). Three factors significantly improved the post-op survival rate during the initial model creation stage: (i) minimizing myocardial damage and blood loss, (ii) relieving lung congestion during the recovery period of the surgery, and (iii) minimizing the number of ablation attempts to achieve CAVB. To avoid unintended damage of the surrounding myocardium during the electrosurgical energy delivery, the needle shaft was wrapped with an insulating tape exposing only the needle tip. The fine-tipped ablation needle also allowed a partial right thoracotomy rather than midline/median sternotomy which is associated with high mortality and morbidity post-op^20,21,48,49^. We observed increased airway resistance in the CAVB animals during the recovery period likely due to excessive pulmonary secretion caused by the sudden drop in cardiac output. Suppressing pulmonary acute mucous secretion with atropine as described above improved post-op survival rate of the CAVB rats during the initial model creation stage.

### Telemetry recording of ECGs in ambulatory animals with CAVB

Ambulatory ECGs were recorded continuously by implanting a dual-lead, biopotential telemeter (TR50BB, Millar Inc., Houston, TX) in the abdominal cavity according to the manufacturer’s instructions. Lead I was obtained by positioning the electrodes on the left and right latissimus dorsi muscles. Lead II was obtained by positioning the electrodes between the suprasternal fossi and left lower thoracic cage. The electrical signals were transmitted to SmartPad (TR181, Millar Inc., Houston, TX) and analyzed with LabChart Pro (ADInstruments, Colorado Springs, CO).

### Echocardiographic evaluation of CAVB rats

Echocardiography was performed immediately before the surgery, then serially at one week and one month post-surgery with Vevo 2100 High Frequency ultrasound imaging system (VisualSonics, Toronto, Ontario) and analyzed with Vevo Imaging Workstation. Inter-ventricular septal thickness (IVS), LV posterior wall thickness (LVPW), and LV internal diameter (LVID) were measured in short axis during systolic and diastolic phases on M mode imaging. From these measurements, LV end diastolic volume (LVEDV), LV end systolic volume (LVESV), LV fractional shortening (LVFS), LV ejection fraction (LVEF), and approximated LV myocardium mass were calculated.

### Arrhythmia induction by programmed electrical stimulation combined with pharmacologic challenge

Ventricular arrhythmias were induced on five CAVB rats one month after CAVB creation. Animals were anesthetized with 5% isoflurane and placed on a mechanical ventilator after intubation. Anesthesia was maintained with 2% isoflurane during programmed electrical stimulation (PES). The right jugular vein of the anesthetized CAVB rat was cannulated with a 21-gauge polyethylene tubing which was navigated into the right ventricle. Using the polyethylene tubing as a guide sheath, a platinum wire was inserted through the lumen of the tubing into the right ventricular cavity until ventricular electrical potentials could be detected. PES was performed with current stimulus isolator (FE180, ADInstruments, Colorado Springs, CO) with or without isoproterenol (5 mg/kg, ip) following our previous protocol^29^. Pacing thresholds were determined, and stimulation was delivered at a 1 ms pulse width at twice the capture threshold. An S1 drivetrain of 10 stimuli (100 ms interval) was applied followed by an extra stimulus (S2) starting at a coupling interval of 80 ms and by 1 ms decrements until the effective refractory period was reached. Another extra stimulus (S3) followed S2, decremented by 1 ms until the ventricular effective refractory period was reached. After this, a train of programmed stimulation with three extra stimuli after S1 (S2–S4) was performed until a ventricular arrhythmia was induced. If the rat failed to develop ventricular arrhythmia with 3 extra stimuli (S1–S4), the animal was deemed non-inducible.

### Histopathology

The heart from seventeen rats were harvested, fixed in 10% formalin for 24 hours and paraffin-embedded for histopathological examination of the AVN region. The animals were grouped as follows: 3-month old normal male (n = 3), 3-month old normal female (n = 3), 6-month old normal female (n = 3), 6-month old normal male (n = 3) and 6-month old female with CAVB (n = 5). The paraffin-embedded tissue samples were sectioned at 5 µm thickness. Masson’s trichrome staining was performed to examine the native AVN structure and subsequent tissue necrosis post-electrosurgical energy delivery. Staining was performed according to manufacturer’s protocol (Masson’s Trichrome Staining Kit #KTMTR, American MasterTech, Inc., Lodi, CA). Stained sections were scanned with Hamamatsu Nanozoomer (Hamamatsu Photonics K.K., Japan). We measured the fibrotic area from upper margin of IVS to lower margin of IAS including the annulus ring of the mitral valve. The mitral valve leaflet was not included. Fibrotic area was measured by manually drawing a region of interest (ROI) around the circumference of blue collagen tissue. ROI area was measured using NDP.view2 software.

### Direct injection of TBX18 into the left ventricular apex of CAVB rat *in vivo*

Upon creating CAVB, the animals were allowed to recover from the right thoracotomy and monitored for one week to confirm stable CAVB. Upon validating stable CAVB, the animals underwent a partial left thoracotomy in the fourth intercostal space to deliver an adenoviral vector expressing either *TBX18*-IRES-zsGreen1 (n = 4) or GFP (n = 4) to the heart. For each animal, 1 × 10^8^ fluorescence forming units (ffu) of the adenoviral vector was injected in 100 µl of volume into the left ventricular apex of the heart. A telemetry device was implanted immediately after injection of the viral vector.

### Statistics

All data are expressed as mean ± standard deviation (SD) at a 95% confidence limit. For longitudinal comparison of continuous variables such as mean heart rates, weight, and echocardiographic parameters, the statistical significance was tested using repeated-measures ANOVA with subsequent Bonferroni multiple comparisons test. Statistical analysis was performed using SPSS version 23.0 (IBM, Chicago, IL).

## Supplementary information

Supplementary figure 1

Supplementary video 1

Supplementary video 2

## Data Availability

We will make available complete protocols for instrumentation of the surgery as well as the surgery protocol itself without undue qualifications in the MTAs. All published data will be shared promptly. The rodents harboring complete atrioventricular block generated from the disclosed surgical protocol are not transferrable due to the material and personnel expenses that are associated in creating the animal model.
