# Supplementary material for: A rat model of complete atrioventricular block recapitulates clinical indices of bradycardia and provides a platform to test disease-modifying therapies
Source: Sci Rep. 2019 May 6;9:6930. doi: 10.1038/s41598-019-43300-9 (PMC6502940; doi:10.1038/s41598-019-43300-9)
Supplement: Supplementary file 1 — Supplementary figure 1 [file 41598_2019_43300_MOESM1_ESM.pdf]

A rat model of complete atrioventricular block recapitulates clinical indices of bradycardia and provides a platform to test disease-modifying therapies

Nam Kyun Kim, MD[1,3], David Wolfson, BS[2], Natasha Fernandez, BS[1], Minji Shin, BS[1] and Hee Cheol Cho, PhD[1,2]

1Department of Pediatrics, Emory University, Atlanta, GA

2Department of Biomedical Engineering, Georgia Institute of Technology and Emory University, Atlanta, GA

3Department of Pediatrics, Yonsei University College of Medicine, Seoul, South Korea

Supplementary Figure 1

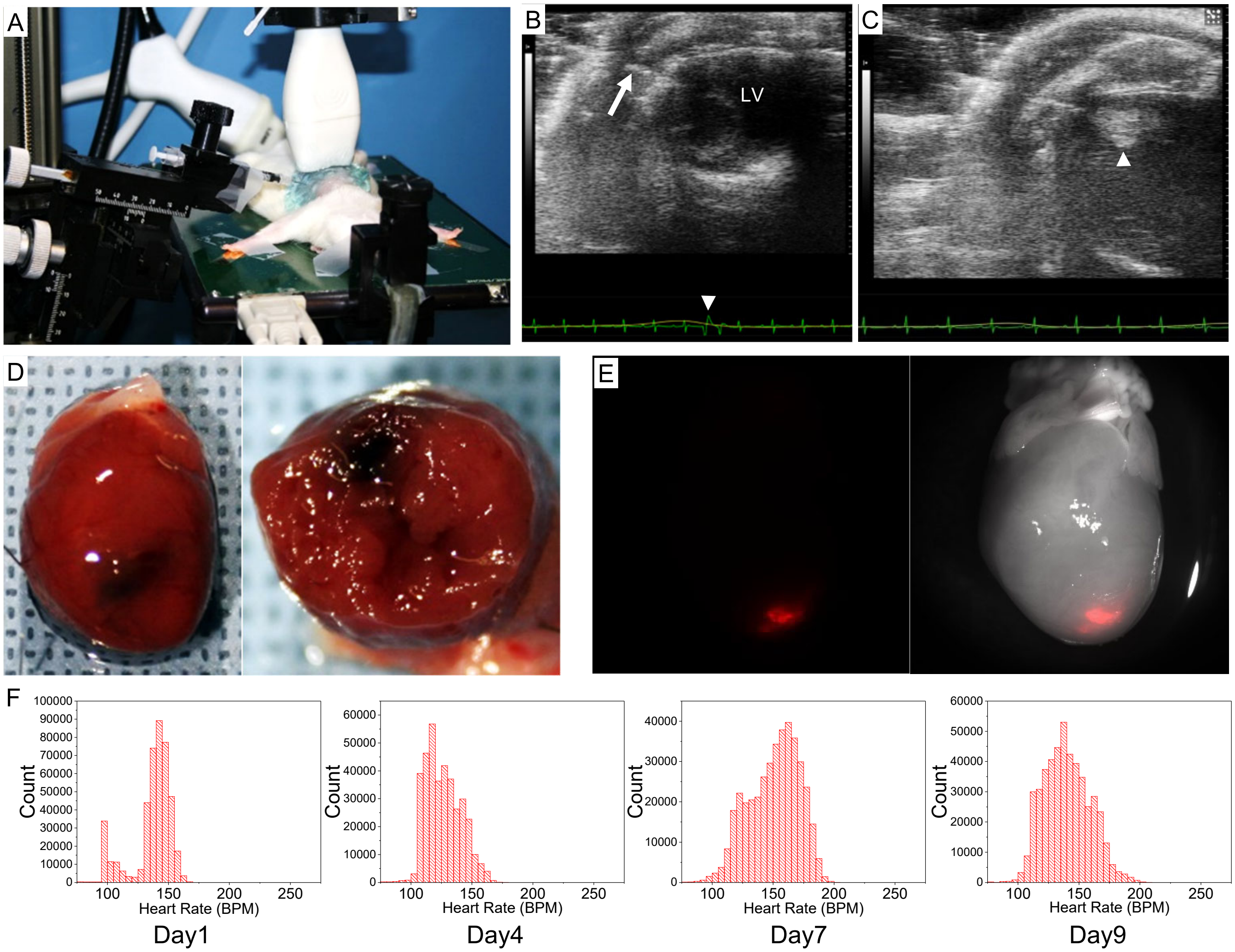

Supplementary Figure 1. Echocardiography-guided, direct myocardial gene delivery through the chest wall in the adult rat.

A. A 27G injection needle on a syringe was maneuvered with a micromanipulator that was stationed on the echocardiogram stage. B. An echocardiographic image during advancing of an injection needle (arrow) through the chest wall. Needle insertion into the myocardium elicited a PVC (an arrowhead). C. An echocardiographic image illustrates the biologic injected as a bolus into the left ventricular myocardium (an arrowhead). D. The heart was harvested 30 min after direct injection of Trypan Blue. E. In another rat, Adeno-mCherry (Vector Biolabs, cat.#1767) was injected. The heart was harvested 4 days after *in vivo* injection for imaging, showing successful expression of the red fluorescent protein (left image) in the left ventricular wall, as evidenced by the merged bright field and fluorescence image (right image). F. Beat-to-beat histograms showing the distribution of beating rates during the first week after CAVB induction in rats.
